# The Incidence, Clinical Outcomes, and Risk Factors of Thrombocytopenia in Intra-Abdominal Infection Patients: A Retrospective Cohort Study

**DOI:** 10.1371/journal.pone.0147482

**Published:** 2016-01-25

**Authors:** Qin Wu, Jianan Ren, Gefei Wang, Guanwei Li, Guosheng Gu, Xiuwen Wu, Yuan Li, Jun Chen, Yunzhao Zhao, Jieshou Li

**Affiliations:** Department of General Surgery, Jinling Hospital, Medical School of Nanjing University, Nanjing, China; The University of Hong Kong, HONG KONG

## Abstract

**Background:**

Studies on the incidence and risk factors of thrombocytopenia among intra-abdominal infection patients remain absent, hindering efficacy assessments regarding thrombocytopenia prevention strategies.

**Methods:**

We retrospectively studied 267 consecutively enrolled patients with intra-abdominal infections. Occurrence of thrombocytopenia was scanned for all patients. All-cause 28-day mortality was recorded. Variables from univariate analyses that were associated with occurrence of hospital-acquired thrombocytopenia were included in a multivariable logistic regression analysis to determine thrombocytopenia predictors.

**Results:**

Median APACHE II score and SOFA score of the whole cohort was 12 and 3 respectively. The overall ICU mortality was 7.87% and the 28-day mortality was 8.98%. The incidence of thrombocytopenia among intra-abdominal infection patients was 21.73%. Regardless of preexisting or hospital-acquired one, thrombocytopenia is associated with an increased ICU mortality and 28-day mortality as well as length of ICU or hospital stay. A higher SOFA and ISTH score at admission were significant hospital-acquired thrombocytopenia risk factors.

**Conclusions:**

This is the first study to identify a high incidence of thrombocytopenia in patients with intra-abdominal infections. Our findings suggest that the inflammatory milieu of intra-abdominal infections may uniquely predispose those patients to thrombocytopenia. More effective thrombocytopenia prevention strategies are necessary in intra-abdominal infection patients.

## Introduction

Intra-abdominal infections (IAIs), defined as infections occurring within an abdominal organ or in the abdominal cavity, are a frequent and dangerous entity in the treatment of critically illness patients.[1] Although the therapeutic techniques including pharmaceutical and radiographic interventions develop a lot, the overall mortality in patients with IAIs remains to exceed 10% and IAI brings a substantial burden for patients, surgeons, and the healthcare system.[2, 3]

Independent of the cause, patients with IAIs are at high risk of severe complications. Thrombocytopenia is one of the very common laboratory abnormalities among IAI patients.[4, 5] The underlying pathogenesis of thrombocytopenia in IAI remains incompletely understood, but is thought to be the result of multiple factors, including immobility, activation of thrombo-inflammatory pathways, disseminated intravascular coagulation (DIC), and venous stasis.[5–7]

Recently, the epidemiology of thrombocytopenia has been investigated by several studies as well as the impact of thrombocytopenia on outcomes among critically illness patients[8]. However, depending on its definition and the category of patients in which it was studied, the prevalence and incidence of thrombocytopenia in ICU varied a lot.[5, 9] Most of those well-conducted studies were performed in more heterogeneous groups of critically illness patients.[5, 10] To date, there remain no studies investigating thrombocytopenia incidence, clinical outcome and risk factors specifically in IAI patients. This absence of data limits advances in the prevention of thrombocytopenia among these IAIs patients. Thus, identification of the incidence, the impact on the outcome and risk factors of thrombocytopenia in IAI patients remains paramount. The purpose of this study was to retrospectively determine the incidence, clinical characteristics, outcomes and predictors of thrombocytopenia in a large cohort of IAI patients.

## Method

### Study Population

This retrospective database cohort study was conducted at Jinling Hospital, Nanjing, China. The Jinling Hospital is a national tertiary academic medical center for the treatment of gastrointestinal fistula. The study was approved by the Institutional Review Board of Jinling Hospital and informed consent was not required because of the anonymous and the observational nature of the study. Consecutive adult admissions from January 1, 2013 to December 31, 2014 were identified from a prospectively collected hospital database via individual chart review. Patient database information at our hospital is either collected automatically by computer or is entered by physicians. Specialized software (Haitai Software, Nanjing Haitai Medical Information System Company, Nanjing, China) is used to monitor integrity of the data.

### Study Protocol

All patients admitted during the study period were scanned for the diagnosis of IAI according to the medical records. IAI in this study was defined as: (1) Patient has organisms cultured from purulent material from intra-abdominal space obtained during a surgical operation or needle aspiration; (2) Patient has abscess or other evidence of intra-abdominal infection seen during a surgical operation or histopathologic examination; (3) Patient has at least two of the following signs or symptoms with no other recognized cause: fever, nausea, vomiting, abdominal pain, or jaundice and at least one of the following: (a) Organisms cultured from drainage from surgically placed drain; (b) Organisms seen on Gram stain of drainage or tissue obtained during surgical operation or needle aspiration; (c) Organisms cultured from blood and radiographic evidence of infection. [11]

For patients with IAI, the occurrence of thrombocytopenia is determined. In this study, thrombocytopenia was defined as a peripheral platelet count < 100×10^9^/L, or a relative platelet count drop of > 50% from baseline. Patients whose age were less than 18 years at admission were excluded.

Based on platelet counts at admission, patients were divided into a preexisting thrombocytopenia group and a non-preexisting thrombocytopenia group. For patients without thrombocytopenia at admission, records of platelet counts during hospitalization were reviewed to identify any episodes of thrombocytopenia. According to the platelet count during hospitalization, patients without preexisting thrombocytopenia were further categorized as hospital-acquired thrombocytopenia group and non-hospital-acquired thrombocytopenia group.

To explore the risk factor of thrombocytopenia, database records of patients were reviewed. Using the DIC scoring system of the Japanese Association for Acute Medicine DIC scoring system (JAAM score) and the DIC score of the International Society of Thrombosis and Haemostasis (ISTH score), we determined if any episodes of DIC occurred during thrombocytopenia in our department.

### Data collection

In our hospital, laboratory test, including routine analysis of blood would be done within 4 h after admission for all patients to establish the baseline. Data recorded routinely on admission also included age, gender, primary and secondary admission diagnoses, and surgical procedures preceding admission. The Acute Physiology and Chronic Health Evaluation II (APACHE II) score, the Sequential Organ Failure Assessment (SOFA) score, the JAAM score and the ISTH score were calculated by the attending physician who was in charge of the patient. After admission, venous blood for all laboratory tests, including platelet counts would be drawn whenever required, according to the judgment of the attending physician (available 24 hrs/day). Usually, laboratory values were calculated at least once per day during ICU stay or at least every other day during hospital stay. After obtaining the blood sample, laboratory values were calculated within 2 hours of blood collection. To be specific, platelet counts were measured with the Beckman Coulter LH 750 (Beckman Coulter, Miami, FL, USA). Other laboratory parameters at admission and during hospitalization were collected, including white red blood cell counts, C-reactive protein, blood cell counts, international normalized ratio, prothrombin time, activated partial thromboplastin time, fibrinogen, Alkaline Phosphatase, Alanine aminotransferase, Aspartate amino Transferase, gamma-glutamyl transpeptidase, blood urea nitrogen and electrolytes. Records of bleeding events, mechanical ventilation events, and renal replacement therapy event during hospitalization were extracted.

### Statistical Analyses

Data were analyzed with SPSS Statistics 17 (SPSS Software, Chicago, IL, USA), Origin software (version 3.78; Microcal Software Inc., Northampton, MA) and GraphPad Prism (GraphPad Software, San Diego, CA, USA). Discrete variables are expressed as counts (percentage) and continuous variables as means ± SD or median and interquartile range (IQR) unless stated otherwise. The Kolmogorov—Smirnov test was used to verify the normality of distribution of continuous variables. Categorical variables were compared with the chi-squared test. Continuous variables conforming to a normal distribution were compared using analysis of variance and Student’s t test; otherwise the Kruskal—Wallis and Mann—Whitney U test was applied. Cumulative survival curves were constructed with the Kaplan-Meier method; the log-rank test was used to assess statistical differences between survival curves. To assess the risk factors for hospital-acquired thrombocytopenia and to determine if preexisting or hospital-acquired thrombocytopenia were independent predictors of mortality, multivariate analyses were performed with logistic regression. The level of significance was set at 0.05.

## Results

**Fig 1:**
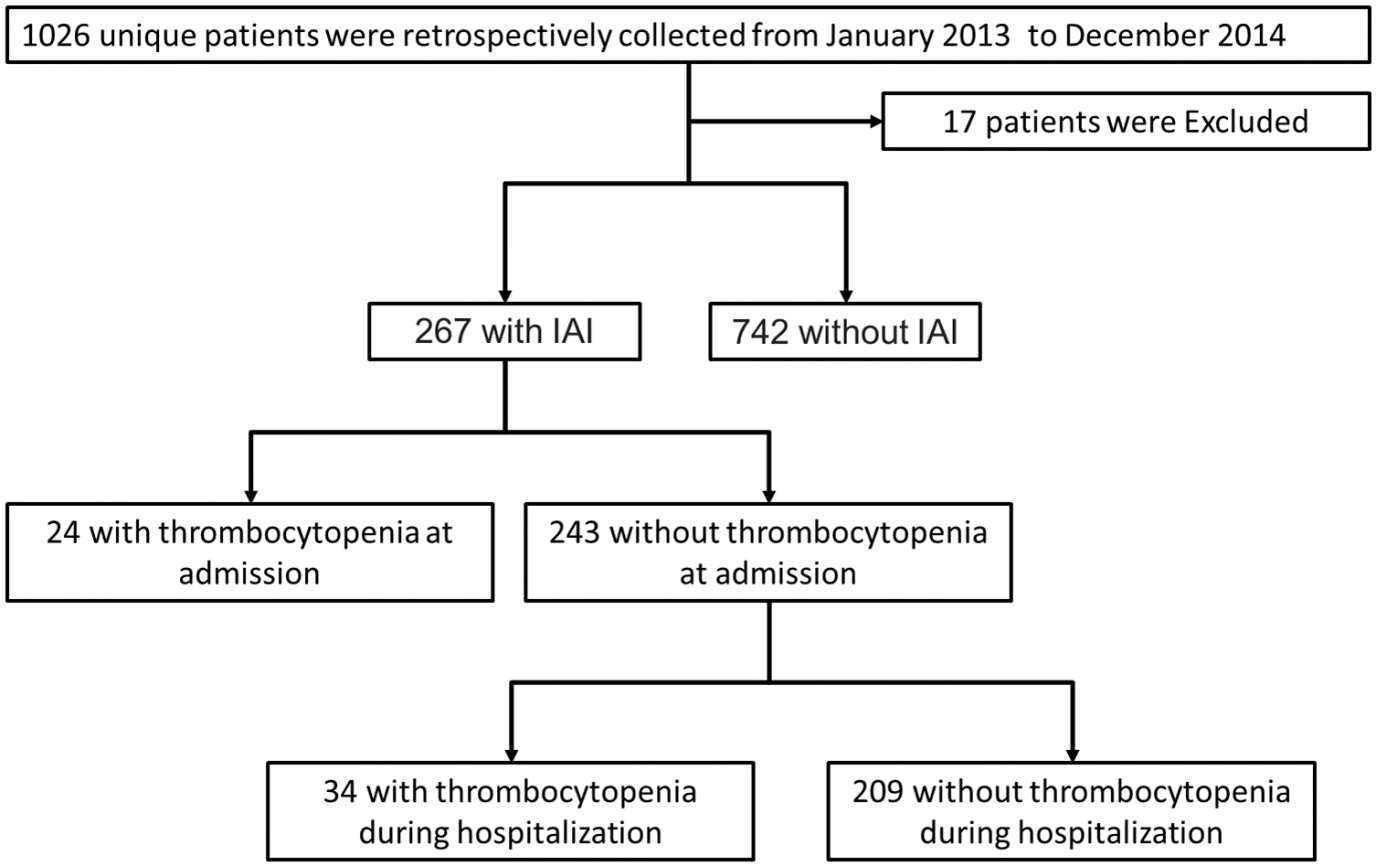
Flowchart of current study. A total of 1,026 unique patients was retrospectively collected and 17 of them were excluded for age was less than 18 years at admission. Among them, 267 patients were diagnosed as IAI. For IAI patients, 24 were admitted with thrombocytopenia. The remaining 243 patients without preexisting thrombocytopenia, 34 experienced at least one episode of thrombocytopenia during hospitalization.

**Fig 2:**
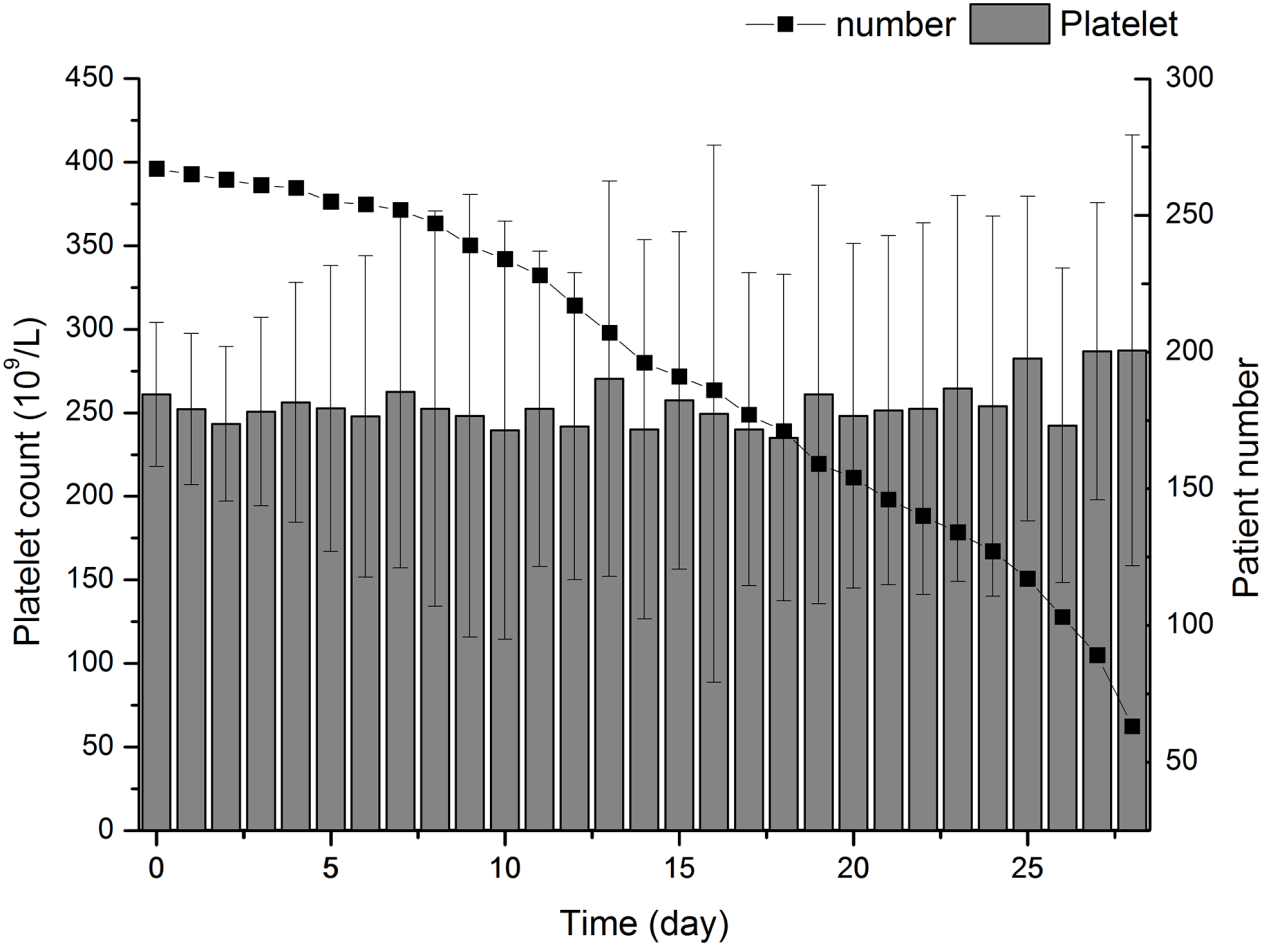
Changing trend of platelet counts from Day0 to Day28. Bar chart representing average platelet counts and patient numbers after admission. Day0 stands for the admission.

A total of 1,026 consecutive patients admitted to our department during the study period were included in. Among them, 17 were excluded because of age was less than 18 year at admission. For the remaining 1,009 patients, 267 patients who admitted as IAI were enrolled in (Fig 1). Table 1 summarized the baseline characteristics of the study population. Accompanied by a male predominance, the mean ages of the whole cohort were 49.45. Surgical complication and traffic accident were the most common primary disease that caused the IAI. The median APACHE II score and SOFA score was 12 and 3, respectively. More than half of the patients were admitted into ICU. The overall ICU mortality was 7.87% and the 28-day mortality was 8.98%. The median ICU length of stay (LOS) was 7 days and the hospital LOS was 29 days. The average platelet counts in enrolled patients within 28 days after admission was demonstrated in Fig 2.

**Table 1:** Demographics, Clinical and Outcome data of all enrolled patients.

Parameters	All enrolled patients (n = 267)
Demographic Data	
Age, mean (SD),y	49.45 (15.89)
Male, n (%)	193 (72.28)
BMI, mean (SD),	20.56 (3.69)
Primary Disease, n (%)	
Traffic accident	38 (14.23)
Injury ^a^	34 (12.73)
Surgical complication ^b^	161 (60.30)
Others	34 (12.73)
Fistula location, n (%)	
Small bowel	74 (27.72)
Colon	56 (20.97)
Duodenum	31 (11.61)
Pancreas	12 (4.49)
Stomach	18 (6.74)
Multiple viscera ^c^	76 (28.46)
Clinical Data (at admission)	
APACHE II Score, median (SD)	12 (4)
SOFA score, median (SD)	3 (1)
ISTH, median (SD)	0 (0)
JAAM, median (SD)	1 (1)
WBC, mean (SD),×10^9^/L	10.31 (6.79)
RBC, mean (SD),×10^9^/L	3.52 (0.76)
PC, mean (SD),×10^9^/L	260.88 (146.76)
CRP, mean (SD), mg/L	67.85 (65.21)
PCT, mean (SD), ng/mL	3.40 (11.29)
INR, mean (SD)	1.18 (0.15)
PT, mean (SD), s	13.60 (1.82)
APTT, mean (SD), s	34.29 (7.45)
Fib, mean (SD), g/L	3.71 (1.25)
Serum albumin, mean (SD),g/L	34.11 (6.18)
ALP, mean (SD),U/L	129.55 (101.78)
ALT, mean (SD),U/L	51.21 (180.55)
AST, mean (SD),U/L	52.73 (184.28)
GGT, mean (SD),U/L	118.54 (141.92)
Bilirubin, mean (SD),umol/L	31.23 (41.57)
Creatinine, mean (SD),umol/L	71.44 (70.62)
BUN, mean (SD), mmol/L	7.16 (6.76)
Serum sodium, mean (SD),mmol/L	138.10 (5.73)
Serum potassium, mean (SD),mmol/L	4.13 (0.70)
Serum chloride, mean (SD),mmol/L	101.76 (5.78)
Serum calcium, mean (SD),mmol/L	2.11 (0.20)
Serum phosphorus, mean (SD),mmol/L	1.30 (0.36)
Mechanical ventilation, n (%)	26 (9.73)
Renal replacement therapy, n (%)	12 (4.49)
Incidence of preexisting thrombocytopenia, n (%)	24 (8.99)
Incidence of hospital-acquired thrombocytopenia, n (%)	34 (13.99)
Incidence of ICU administration, n (%)	144 (53.92)
Reasons for ICU administration, n (%)	
Organ dysfunction	16 (5.99)
Unstable hemodynamic status	15 (5.62)
Post damage control surgery	43 (16.10)
Others	6 (2.25)
Undetermined	64 (23.97)
Outcome Data	
Hospital mortality, n (%)	35 (13.11)
28-day mortality, n (%)	24 (8.98)
ICU mortality, n (%)	21 (7.87)
ICU LOS ^d^, median (IQR),d	7 (9)
ICU LOS ^e^, median (IQR),d	6 (11)
Hospital LOS ^f^, median (IQR),d	29 (30)
Hospital LOS ^g^, median (IQR),d	32 (31)
Hospital cost, median (IQR), dollar	30,837.33 (29,501)

BMI: Body Mass Index; APACHE: Acute Physiology and Chronic Health Evaluation; WBC: white blood cell; RBC: red blood cell; PC: platelet count; CRP: C-reaction protein; INR: International Normalized Ratio; PT: prothrombin time; APTT: activated partial thromboplastin time; Fib: fibrinogen; ALP: Alkaline Phosphatase; ALT: Alanine aminotransferase; AST: Aspartate amino Transferase; GGT: gamma-glutamyl transpeptidase; BUN: blood urea nitrogen; LOS: length of stay; IQR: interquartile range;

^a^ Injury includes gunshot, falling, cuts, bruising

^b^ Patients who developed into intra-abdominal infection after elective surgery were categorized as surgical complication

^c^ For all ICU patients Includes appendicitis, ileus, several nonmalignant processes, pancreatitis, cholecystitis, and vascular disease

^d^ For all ICU patients

^e^ For ICU survivors

^f^ For all patients

^g^ For all survivors

### Preexisting thrombocytopenia at hospital admission

**Fig 3:**
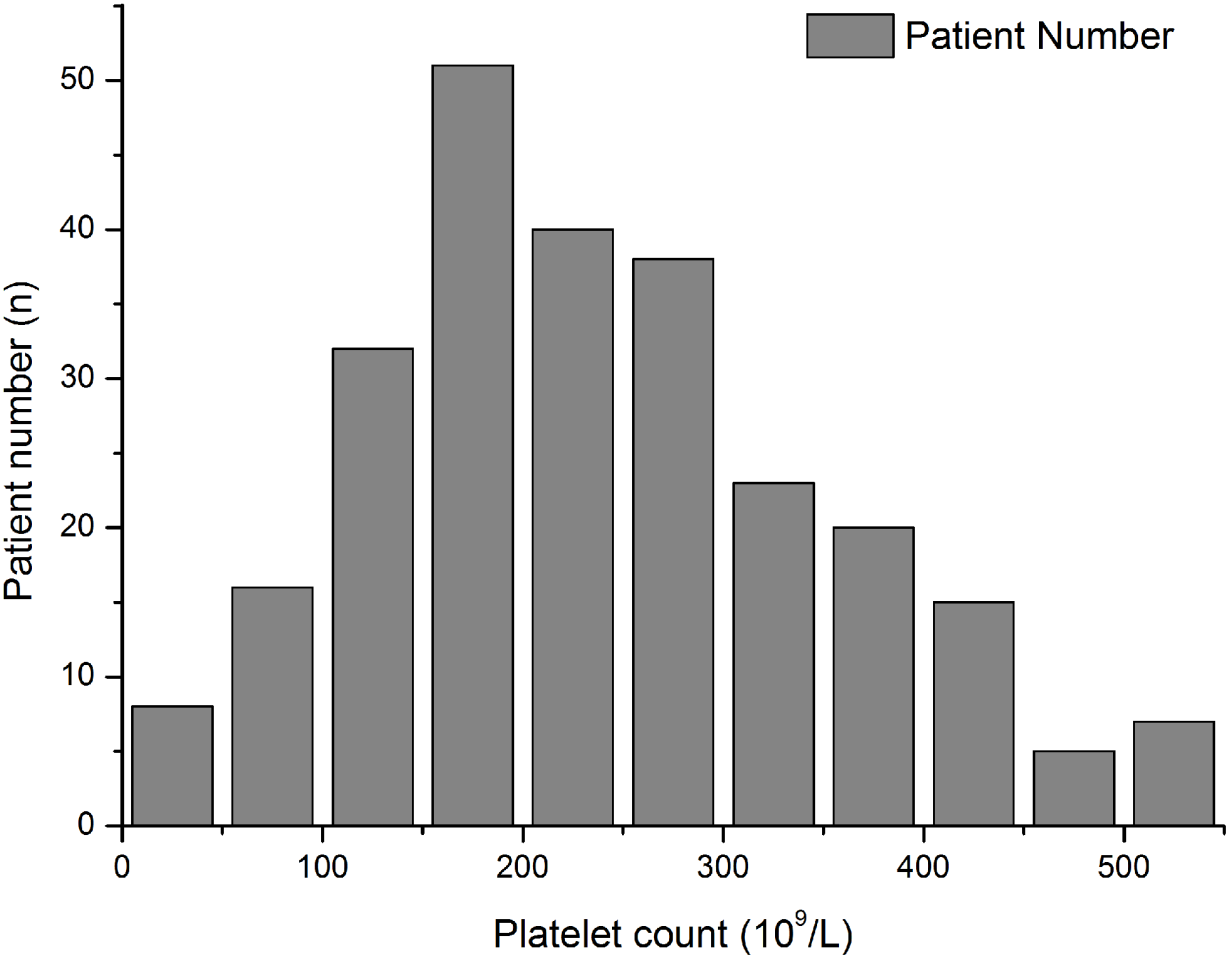
Distribution of platelet counts at admission for all enrolled patients. 24 IAI patients were admitted with preexisting thrombocytopenia.

**Fig 4:**
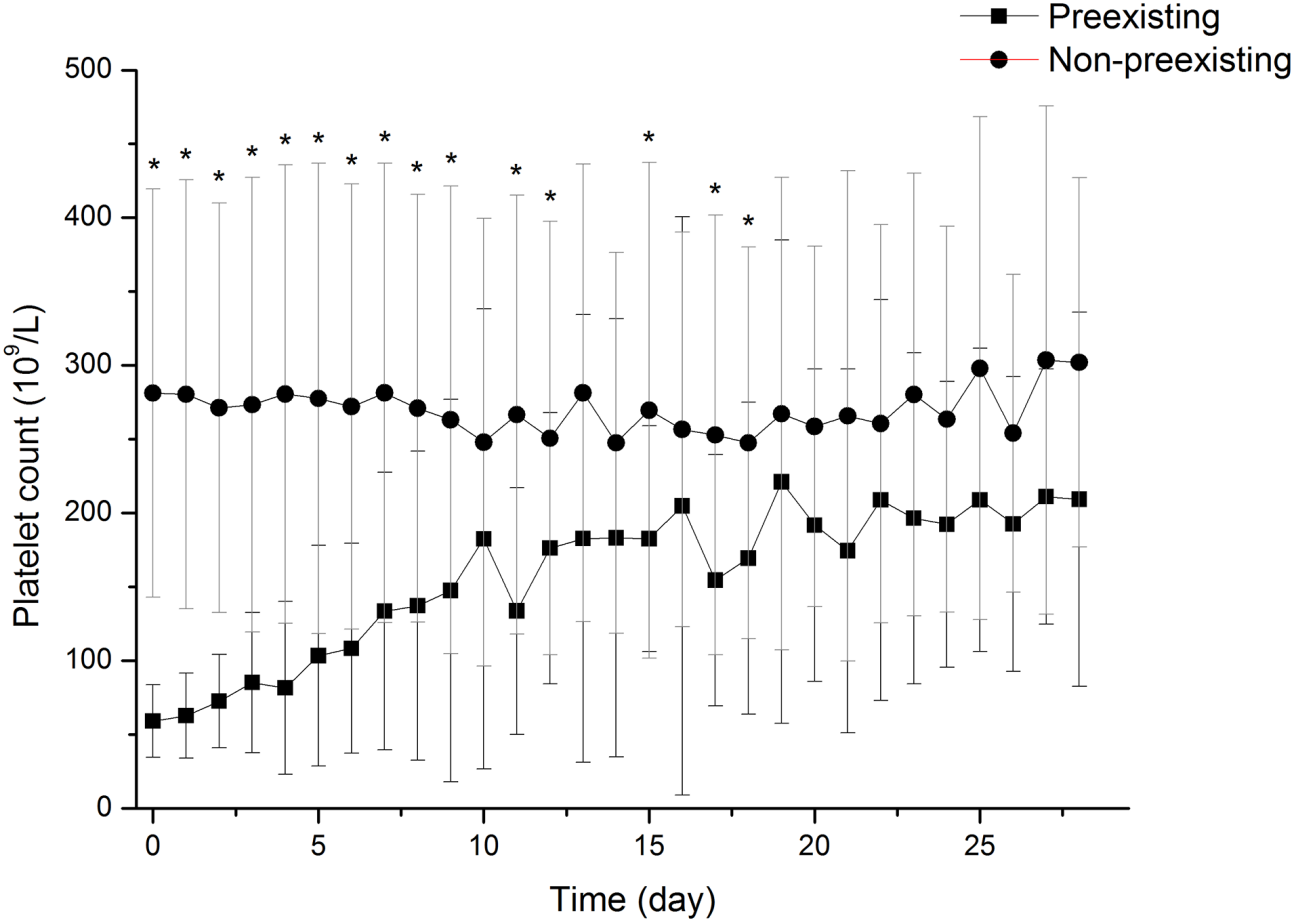
Changing trend of platelet counts in preexisting thrombocytopenic and non-preexisting thrombocytopenic patients after admission over time. A statistically significantly difference was exhibited between preexisting thrombocytopenia group and non-preexisting group after admission. * p<0.05.

Fig 3 showed the distribution of platelet counts of all patients on admission. The incidence of preexisting thrombocytopenia at hospital admission among IAI patients were 8.99% (24 out of 267, Fig 1) while only 1.62% (12/742) non-IAI patients admitted to hospital with preexisting thrombocytopenia. Demographic data of the patients with pre-existing thrombocytopenia had no significant differences compared with those without preexisting thrombocytopenia (Table 2). Fig 4 demonstrated the changing trend of platelet counts in patients with or without preexisting thrombocytopenia after admission. The platelet counts in preexisting thrombocytopenia patients were significantly lower than those without preexisting thrombocytopenia within 7 days after admission. For the outcome data, patients with preexisting thrombocytopenia at admission had higher ICU and 28-day mortality rates, as well as a longer ICU LOS compared with patients without preexisting thrombocytopenia (Table 2). The survival analysis showed the significant difference in 28-day mortality in patients with or without preexisting thrombocytopenia (S1 Fig). In a multivariable logistic regression analysis, occurrence of pre-existing thrombocytopenia was independently associated with a greater risk of 28-day death (OR 4.739; 95% CI 1.038–16.280; p = 0.013, Table 3).

**Table 2:** Characteristics and Outcome of the Study Cohort According to the platelet count at Admission.

Parameters	Preexisting Thrombocypenic patients (n = 24)	Non-preexisting thrombocytopenic patients (n = 243)	P
Demographic Data			
Age, mean (SD),y	55.71 (16.99)	48.84 (15.68)	0.063
Male, n (%)	17 (70.83)	176 (72.43)	0.947
BMI, mean (SD),	19.55 (4.55)	20.65 (3.61)	0.236
Primary Disease, n (%)			0.237
Traffic accident	1 (4.17)	37 (15.22)	
Injury ^a^	2 (8.33)	32 (13.17)	
Surgical complication ^b^	19 (79.17)	142 (58.44)	
Others	2 (8.33)	32 (13.17)	
Clinical Data (at admission)			
APACHE II Score, median (SD)	13 (4.5)	12 (4)	0.142
SOFA score, median (SD)	4.5 (3)	3 (1)	<0.001
ISTH score, median (SD)	0 (0)	0 (0)	0.124
JAAM score, median (SD)	1 (1)	1 (1)	0.126
WBC, mean (SD),×10^9^/L	10.35 (7.79)	10.30 (6.70)	0.971
RBC, mean (SD),×10^9^/L	3.05 (0.73)	3.57 (0.74)	0.003
PC, mean (SD),×10^9^/L	59.16 (24.48)	281.32 (138.25)	<0.001
CRP, mean (SD), mg/L	100.84 (68.62)	64.47 (64.05)	0.022
PCT, mean (SD),ng/mL	17.64 (22.64)	1.55 (5.17)	<0.001
INR, mean (SD)	1.28 (0.17)	1.17 (0.15)	0.004
PT, mean (SD), s	14.86 (1.94)	13.47 (1.77)	0.003
APTT, mean (SD), s	36.90 (7.77)	34.04 (7.40)	0.128
Fib, mean (SD), g/L	2.94 (1.36)	3.79 (1.21)	0.039
Serum albumin, mean (SD),g/L	38.21 (4.67)	34.71 (6.00)	<0.001
ALP, mean (SD),U/L	123.25 (87.21)	130.19 (103.33)	0.722
ALT, mean (SD),U/L	44.91 (33.62)	51.88 (189.73)	0.864
AST, mean (SD),U/L	52.10 (44.87)	52.79 (192.47)	0.971
GGT, mean (SD),U/L	112.35 (137.22)	119.19 (142.74)	0.838
Bilirubin, mean (SD),umol/L	66.99 (69.78)	27.22 (35.19)	0.018
Creatinine, mean (SD),umol/L	79.72 (65.34)	70.57 (71.24)	0.564
BUN, mean (SD), mmol/L	13.81(10.75)	6.46 (5.81)	<0.001
Serum sodium, mean (SD),mmol/L	141.68 (6.65)	137.73 (5.51)	0.013
Serum potassium, mean (SD),mmol/L	3.73 (0.54)	4.17 (0.70)	0.005
Serum chloride, mean (SD),mmol/L	105.84 (8.41)	101.39 (5.34)	0.042
Serum calcium, mean (SD),mmol/L	1.98 (0.21)	2.12 (0.19)	0.010
Serum phosphorus, mean (SD),mmol/L	1.08 (0.28)	1.32 (0.35)	0.001
Mechanical ventilation, n (%)	3 (33.33)	23 (33.74)	0.632
Renal replacement therapy, n (%)	2 (29.17)	10 (31.28)	0.341
Incidence of ICU administration, n (%)	17 (53.92)	127 (53.92)	0.082
Outcome Data			
Hospital mortality, n (%)	24 (100.00%)	11 (6.99)	<0.001
28-day mortality, n (%)	16 (66.67)	8 (3.29)	<0.001
ICU mortality, n (%)	11 (45.83)	10 (4.11)	<0.001
ICU LOS ^c^, median (IQR),d	12 (22.75)	6 (10)	0.030
ICU LOS ^d^, median (IQR),d	15.5 (64.5)	6 (8)	<0.001
Hospital LOS ^e^, median (IQR),d	29 (33)	29 (30)	0.226
Hospital LOS ^f^, median (IQR),d	39 (32.5)	31 (32)	0.859
Hospital cost, median (IQR), dollar	49,850 (48,650.62)	29,753.37 (27,272.6)	<0.001

BMI: Body Mass Index; APACHE: Acute Physiology and Chronic Health Evaluation; SOFA: Sepsis-related Organ Failure Assessment; ISTH: international society of thrombosis and haemostasis; JAAM: Japanese Association for Acute Medicine DIC scoring system; WBC: white blood cell; RBC: red blood cell; PC: platelet count; CRP: C-reaction protein; PCT: procalcitonine; INR: International Normalized Ratio; PT: prothrombin time; APTT: activated partial thromboplastin time; Fib: fibrinogen; ALP: Alkaline Phosphatase; ALT: Alanine aminotransferase; AST: Aspartate amino Transferase; GGT: gamma-glutamyl transpeptidase; BUN: blood urea nitrogen; LOS: length of stay; IQR: interquartile range;

^a^ Injury includes gunshot, falling, cuts, bruising

^b^ Patients who developed into intra-abdominal infection due to elective surgery were categorized as surgical complication

^c^ For all ICU patients

^d^ For ICU survivors

^e^ For all patients

^f^ For all survivors

**Table 3:** Multivariate analysis of preexisting thrombocytopenia and other covariates associated with 28-day mortality.

Variables	Odds Ratio	95% CI		P value
		lower	upper	
Age				
<49	1.000	-	-	-
>=49	1.566	0.616	3.982	0.346
Gender				
Female	1.000	-	-	-
Male	1.451	0.526	4.002	0.472
BMI				
<20.5	1.008	0.417	2.437	0.986
>=20.5	1.000	-	-	-
Primary diagnosis				
Surgical complication	0.983	0.368	2.629	0.973
Non- surgical complication	1.000	-	-	-
APACHE II score at admission				
<12	2.113	0.813	5.488	0.125
>=12	1.000	-	-	-
SOFA score at admission				
<4	0.832	0.340	2.034	0.686
>=4	1.000	-	-	-
ISTH score at admission				
<1	0.370	0.074	1.848	0.225
>=1	1.000	-	-	-
JAAM score at admission				
<1	0.892	0.360	2.207	0.804
>=1	1.000	-	-	-
Occurrence of preexisting thrombocytopenia				
Yes	4.739	1.038	16.280	0.013
No	1.000	-	-	-

95% CI: 95% confidence interval

### Hospital-Acquired Thrombocytopenia

**Fig 5:**
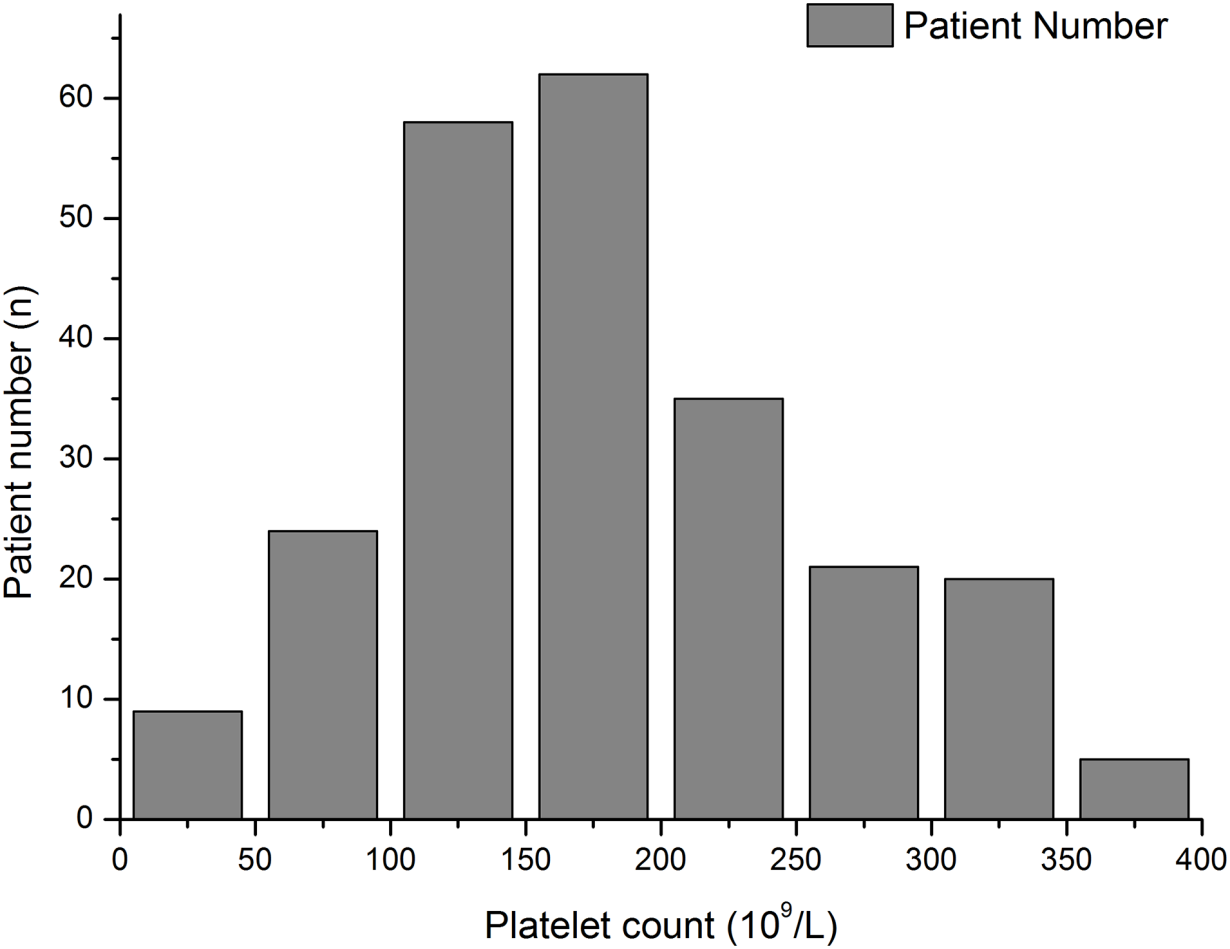
Distribution of the nadir of the platelet count during hospitalization for non-preexisting thrombocytopenic patients. 34 IAI patients non-preexisting thrombocytopenic patients experienced at least one episode of hospital-acquired thrombocytopenia during hospitalization.

**Fig 6:**
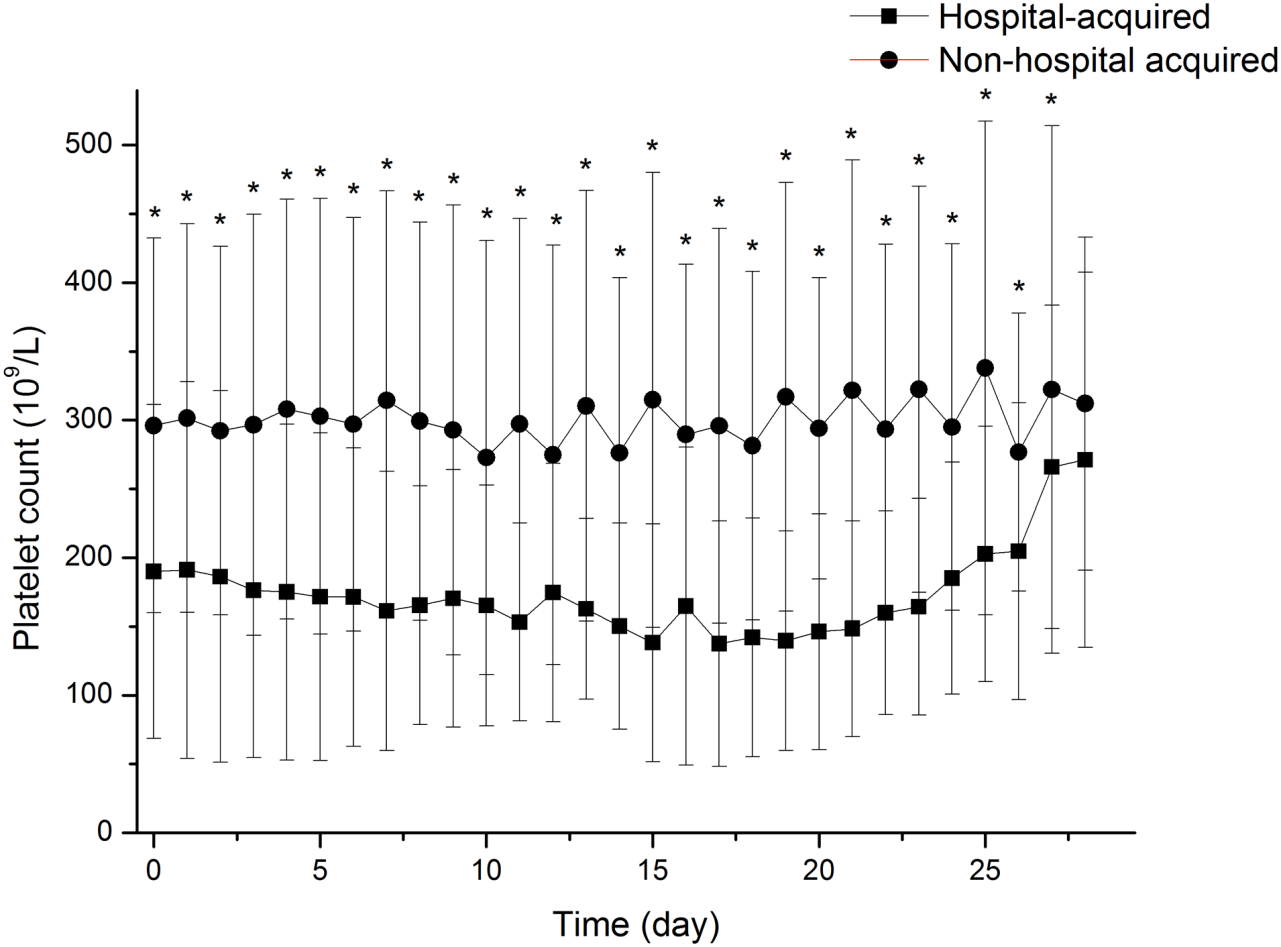
Changing trend of platelet counts in hospital-acquired thrombocytopenic and non- hospital-acquired thrombocytopenic patients after admission over time. A statistically significantly difference was exhibited between hospital-acquired thrombocytopenia group and non- hospital-acquired group after admission. * p<0.05.

For patients without preexisting thrombocytopenia, at least one episode of hospital-acquired thrombocytopenia was occurred in 13.99% (34/243) of the cohort (Fig 1) while only 3.23% (24/742) of non-IAI patients experienced hospital-Acquired Thrombocytopenia. Fig 5 showed the distribution of the nadir of the platelet count during hospitalization in patients without preexisting thrombocytopenia. The changing trend of platelet counts in hospital-acquired and non-hospital-acquired thrombocytopenic patients during hospitalities was demonstrated in Fig 6. Patients with hospital-acquired thrombocytopenia had higher severity scores at admission (Table 4). During hospitalization, platelet counts in hospital-acquired thrombocytopenic patients were significantly lower than those with non-hospital-acquired thrombocytopenic patients. The ICU and 28-day mortality rates in hospital-acquired thrombocytopenic patients were significantly higher and a longer ICU LOS was demonstrated compared to those who did not experience thrombocytopenia throughout their hospital stay. The survival analysis showed the significant difference in 28-day mortality in patients with or without hospital-acquired thrombocytopenia (S2 Fig). In a multivariable logistic regression analysis, the occurrence of hospital-acquired thrombocytopenia was independently associated with 28-day mortality (OR 41.236; 95% CI 11.138–152.669; p < 0.001, Table 5).

**Table 4:** Characteristics and Outcome of the Study Cohort According to the platelet count at Admission.

Parameters	Hospital-acquired Thrombocypenic patients (n = 34)	Non- Hospital-acquired Thrombocypenic patients (n = 209)	P
Demographic Data			
Age, mean (SD),y	53.23 (14.85)	48.12 (15.73)	0.078
Male, n (%)	24 (70.59)	152 (72.73)	0.796
BMI, mean (SD),	20.23 (3.24)	20.72 (3.67)	0.502
Primary Disease, n (%)			0.548
Traffic accident	7 (20.59)	30 (14.35)	
Injury ^a^	6 (17.65)	26 (12.44)	
Surgical complication ^b^	18 (52.94)	124 (59.33)	
Others	3 (8.82)	29 (13.88)	
Clinical Data (at admission)			
APACHE II Score, median (SD)	12 (4)	12 (2.5)	0.780
SOFA score, median (SD)	4 (3)	3 (1)	<0.001
ISTH score, median (SD)	0 (0)	0 (0)	0.059
JAAM score, median (SD)	0 (1)	1 (1)	0.623
WBC, mean (SD),×10^9^/L	9.99 (5.73)	10.35 (6.86)	0.777
RBC, mean (SD),×10^9^/L	2.92 (0.69)	3.68 (0.70)	<0.001
PC, mean (SD),×10^9^/L	190.00 (114.76)	296.08 (136.27)	<0.001
CRP, mean (SD), mg/L	84.39 (62.87)	61.03 (63.78)	0.053
PCT, mean (SD),ng/mL	2.29 (3.61)	1.42 (5.42)	0.471
INR, mean (SD)	1.27 (0.16)	1.15 (0.14)	<0.001
PT, mean (SD), s	14.69 (1.87)	13.26 (1.66)	<0.001
APTT, mean (SD), s	38.03 (11.57)	33.29 (6.09)	<0.001
Fib, mean (SD), g/L	3.15 (1.08)	3.90 (1.20)	<0.001
Serum albumin, mean (SD),g/L	32.34 (6.43)	35.15 (5.84)	0.013
ALP, mean (SD),U/L	132.61 (133.97)	129.72 (96.74)	0.885
ALT, mean (SD),U/L	34.50 (27.56)	55.10 (206.13)	0.574
AST, mean (SD),U/L	38.14 (23.32)	56.45 (215.00)	0.699
GGT, mean (SD),U/L	95.13 (123.31)	123.92 (145.98)	0.296
Bilirubin, mean (SD),umol/L	29.34 (41.94)	24.99 (33.49)	0.043
Creatinine, mean (SD),umol/L	116.93 (150.54)	61.93 (37.79)	<0.001
BUN, mean (SD), mmol/L	9.12 (8.64)	5.97 (5.00)	0.004
Serum sodium, mean (SD),mmol/L	137.13 (5.69)	137.84 (5.49)	0.469
Serum potassium, mean (SD),mmol/L	3.99 (0.78)	4.20 (0.68)	0.974
Serum chloride, mean (SD),mmol/L	101.36 (5.92)	101.39 (5.25)	0.042
Serum calcium, mean (SD),mmol/L	2.04 (0.17)	2.14 (0.19)	0.030
Serum phosphorus, mean (SD),mmol/L	1.19 (0.44)	1.35 (0.33)	0.020
Mechanical ventilation, n (%)	2 (8.33)	20 (9.57)	0.845
Renal replacement therapy, n (%)	2 (8.33)	8 (3.83)	0.302
Incidence of ICU administration, n (%)	30 (88.24)	97 (47.55)	<0.001
Outcome Data			
Hospital mortality, n (%)	24 (100.00%)	11 (6.99)	<0.001
28-day mortality, n (%)	10 (29.41)	6 (2.87)	<0.001
ICU mortality, n (%)	11 (45.83)	4 (1.91)	<0.001
ICU LOS ^c^, median (IQR),d	13 (21.25)	5 (7)	0.030
ICU LOS ^d^, median (IQR),d	12.5 (28.25)	5 (6)	<0.001
Hospital LOS ^e^, median (IQR),d	30 (42.5)	29 (30)	0.226
Hospital LOS ^f^, median (IQR),d	47 (56)	30 (30)	0.859
Hospital cost, median (IQR), dollar	54,497.55 (33,022.63)	33,183.67 (23,037.21)	<0.001

BMI: Body Mass Index; APACHE: Acute Physiology and Chronic Health Evaluation; SOFA: Sepsis-related Organ Failure Assessment; ISTH: international society of thrombosis and haemostasis; JAAM: Japanese Association for Acute Medicine DIC scoring system; WBC: white blood cell; RBC: red blood cell; PC: platelet count; CRP: C-reaction protein; PCT: procalcitonine; INR: International Normalized Ratio; PT: prothrombin time; APTT: activated partial thromboplastin time; Fib: fibrinogen; ALP: Alkaline Phosphatase; ALT: Alanine aminotransferase; AST: Aspartate amino Transferase; GGT: gamma-glutamyl transpeptidase; BUN: blood urea nitrogen; LOS: length of stay; IQR: interquartile range;

^a^ Injury includes gunshot, falling, cuts, bruising

^b^ Patients who developed into intra-abdominal infection due to elective surgery were categorized as surgical complication

^c^ For all ICU patients

^d^ For ICU survivors

^e^ For all patients

^f^ For all survivors

**Table 5:** Multivariate analysis of hospital acquired thrombocytopenia and other covariates associated with mortality.

Variables	Odds Ratio	95% CI		P value
		lower	upper	
Age				
<49	0.773	0.235	2.524	0.671
>=49	1.000	-	-	-
Gender				
Female	1.000	-	-	-
Male	1.202	0.294	4.908	0.798
BMI				
<20.5	0.634	0.185	2.178	0.469
>=20.5	1.000	-	-	-
Primary diagnosis				
Surgical complication	1.954	0.178	21.510	0.584
Non- surgical complication	1.000	-	-	-
APACHE II score at admission				
<12	1.000	-	-	-
>=12	3.161	0.929	10.754	0.065
SOFA score at admission				
<4	1.000	-	-	-
>=4	1.055	0.305	3.656	0.932
ISTH score at admission				
<1	1.000	-	-	-
>=1	1.387	0.291	4.241	0.801
JAAM score at admission				
<1	1.000	-	-	-
>=1	2.360	0.694	8.019	0.169
Occurrence of hospital-acquired thrombocytopenia				
Yes	41.236	11.138	152.669	<0.001
No	1.000	-	-	-

95% CI: 95% confidence interval

In order to predict the occurrence of hospital-acquired thrombocytopenia in patients without preexisting thrombocytopenia at admission, we used logistic regression analysis to determine the risk factors for hospital-acquired thrombocytopenia (Table 6). In particular, SOFA score and ISTH score at admission is associated with the occurrence of hospital-acquired thrombocytopenia.

**Table 6:** Logistic regression analysis of risk factors for developing hospital-acquired thrombocytopenia in patients without preexisting thrombocytopenia.

Variables	Odds Ratio	95% CI		P value
		lower	upper	
Age	0.919	0.315	2.683	0.877
Gender	1.021	0.988	1.055	0.210
BMI	0.955	0.849	1.074	0.440
Primary diagnosis	0.796	0.481	1.317	0.374
APACHE II score at admission	0.925	0.779	1.099	0.376
SOFA score at admission	1.754	1.160	2.651	0.008
ISTH score at admission	6.209	1.563	24.663	0.009
JAAM score at admission	0.669	0.275	1.624	0.374

Primary diagnoses were categorized as trauma, surgical complication, IBD and others.

95% CI: 95% confidence interval

## Discussion

In this study, we retrospectively investigated thrombocytopenia incidence, outcomes, and risk factors in intra-abdominal infection patients. The principal findings were that both preexisting and hospital-acquired thrombocytopenia were common among patients diagnosed as intra-abdominal infection. The occurrence of thrombocytopenia, regardless the preexisting or the hospital-acquired one, was associated with worse outcomes and a longer hospital or ICU LOS. Risk factor analysis indicated SOFA score and ISTH score at admission could be a predictor of hospital-acquired thrombocytopenia for patients without preexisting thrombocytopenia. Although some other studies have investigated the epidemiology of thrombocytopenia in surgical critical care patients[12], to our knowledge, this is the first study to investigate the epidemiology of thrombocytopenia and its possible impact on adverse outcomes in a large cohort of IAI patients.

IAIs are frequent and dangerous entity in ICU. Mortality and morbidity of IAIs are high, causes are numerous, and treatment options are varied.[3, 11] The intensivist is challenged to recognize and treat IAIs in a timely fashion to prevent complications including thrombocytopenia. Thrombocytopenia, which is one of the most commonly observed laboratory abnormalities in ICU population, has an incidence ranging from 13.0% to 44.1%, depending on the study population, the timing and frequency of platelet monitoring, and the definition of thrombocytopenia.[13, 14] Although the epidemiology of thrombocytopenia has been demonstrated in a varied ICU patient population, the incidence of thrombocytopenia is IAI patients is still unknown. Moreover, the association between the occurrence of thrombocytopenia and mortality has not been determined in IAI patients yet.

Our findings identify that IAI patients have a high incidence of preexisting thrombocytopenia and hospital-acquired thrombocytopenia. As a clinically significant phenomenon that is common among critically ill patients, the incidence of thrombocytopenia varies among different study population. Using the same threshold of platelet count, the incidence of preexisting thrombocytopenia ranges from 8.3% to 67.6% among critical illness patients and the incidence of hospital-acquired thrombocytopenia is reported from 13% to 44.1%.[15, 16] In our study, we found that in incidence of preexisting thrombocytopenia and hospital-acquired thrombocytopenia in IAI patients is 8.99% and 13.99% respectively, which is similar to others’ findings. The relatively low incidence of both preexisting thrombocytopenia and hospital-acquired thrombocytopenia may be related to the expectedly lower severity of illness in IAI patients than other surgical ICU patients.

Our data characterize demographic data associated with preexisting thrombocytopenia and hospital acquired thrombocytopenia in IAI patient population. Preexisting thrombocytopenic patients had higher severity scores and more likely elder compared to those without preexisting thrombocytopenia. Anemia and disorder of electrolytes are significantly prevalent among preexisting thrombocytopenic patients, with a higher CRP and PCT level. It indicates that platelet may have a complex interaction with the immune system during infection. For hospital-acquired thrombocytopenia patients, they have a higher SOFA score and also more likely elder compared to others. The disorders of coagulation at admission are significantly more common in hospital-acquired thrombocytopenia patients. It raises the possibility that most of the hospital-acquired thrombocytopenia is not really “hospital acquired” but is derived from implicit coagulation disorders at admission. Still, more studies are needed to confirm our speculation.

We found that either preexisting thrombocytopenia or hospital-acquired thrombocytopenia is associated with mortality. The association between thrombocytopenia and mortality has been well established in mixed critical illness patients. We confirmed the associations in IAI patients. Moreover, our multivariate analysis indicates that the hospital-acquired thrombocytopenia has a more severe negative impact on the mortality compared with the pre-existing thrombocytopenia. Since the occurrence of preexisting thrombocytopenia can hardly be interrupted, hospital-acquired thrombocytopenia may be more clinically important than the preexisting one. The future study should focus on the intervention to prevent the hospital-acquired thrombocytopenia, which may bring a direct improvement of mortality in IAI patients.

The risk factor for developing hospital-acquired thrombocytopenia has been evaluated in our study. A higher SOFA score and ISTH score at admission are associated the occurrence of hospital-acquired thrombocytopenia. This finding indicates that various factors were involved in the process.

This study has several important limitations. In our study cohort, we only investigated the platelet count within 28 days after admission. In 2013, we had conducted a prospective study to evaluate the efficiency of recombinant human thrombopoietin, which has a direct effect on platelet count.[17] Confounding factors may bring in although administration of thrombopoietin in IAI patients has a limited impact on the occurrence of thrombocytopenia. Because of the study design, making a diagnosis of the cause of thrombocytopenia is extremely challenging and the causes of thrombocytopenia were not fully determined. We did not elaborate on relevant therapeutics, such as antibiotics administration, because of the retrospective nature of the study. As with all retrospective database studies, there are concerns about observation bias. Despite these weaknesses, this study does provide an important and novel evaluation of thrombocytopenia in IAI patients.

## Supporting Information

S1 Fig Survival Analysis. A significant difference in mortality was observed between preexisting thrombocytopenia patients and non-preexisting thrombocytopenia patients.
(TIF)

S2 Fig Survival Analysis. A significant difference in mortality was observed between hospital-acquired thrombocytopenia patients and non- hospital-acquired thrombocytopenia patients.
(TIF)
