# Supplementary figures and images for: The Incidence, Clinical Outcomes, and Risk Factors of Thrombocytopenia in Intra-Abdominal Infection Patients: A Retrospective Cohort Study
Source: PLoS One. 2016 Jan 25;11(1):e0147482. doi: 10.1371/journal.pone.0147482 (PMC4725751; doi:10.1371/journal.pone.0147482)

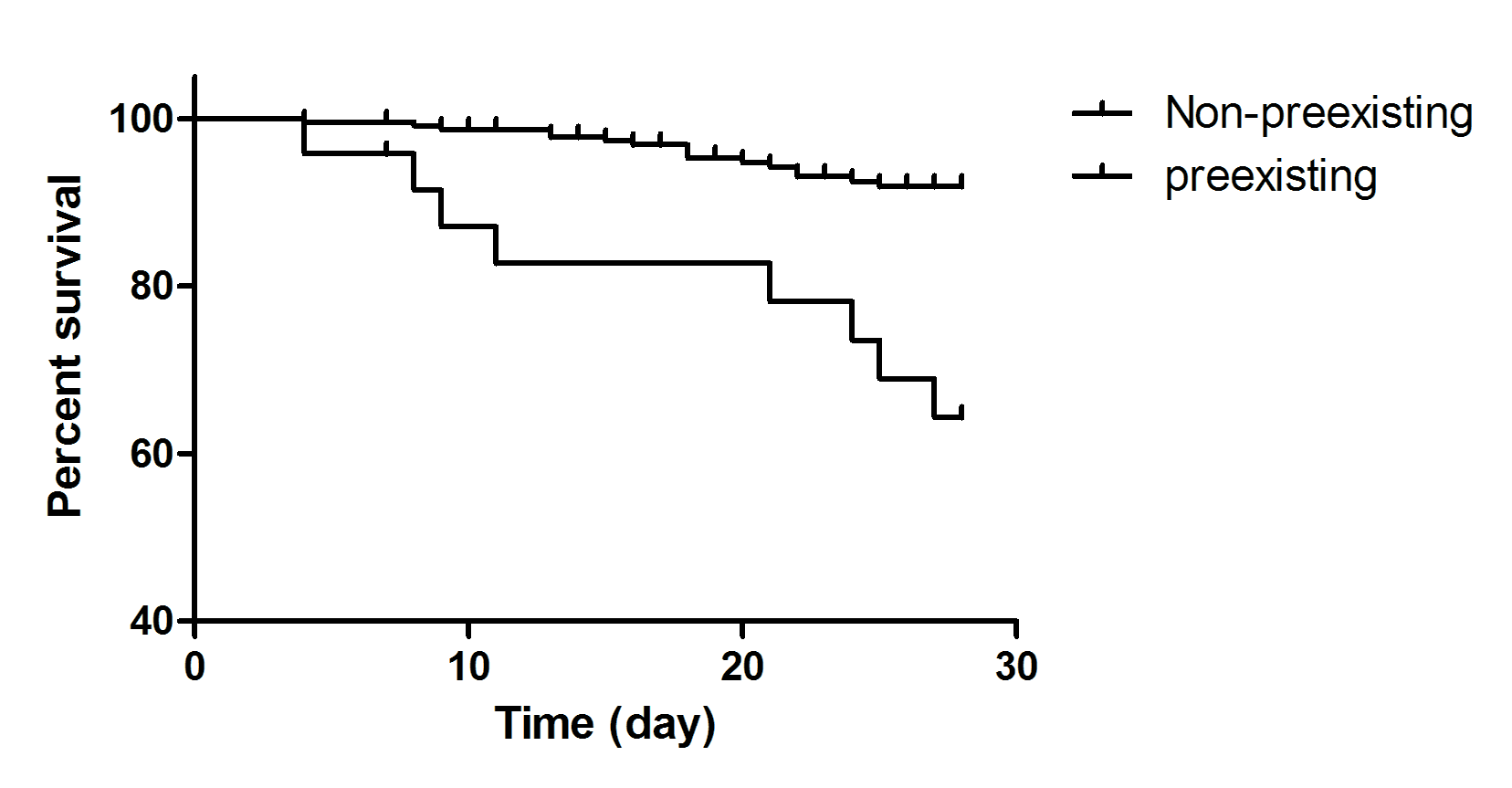

Supplement: S1 Fig — A significant difference in mortality was observed between preexisting thrombocytopenia patients and non-preexisting thrombocytopenia patients. (TIF) [file pone.0147482.s001.tif]

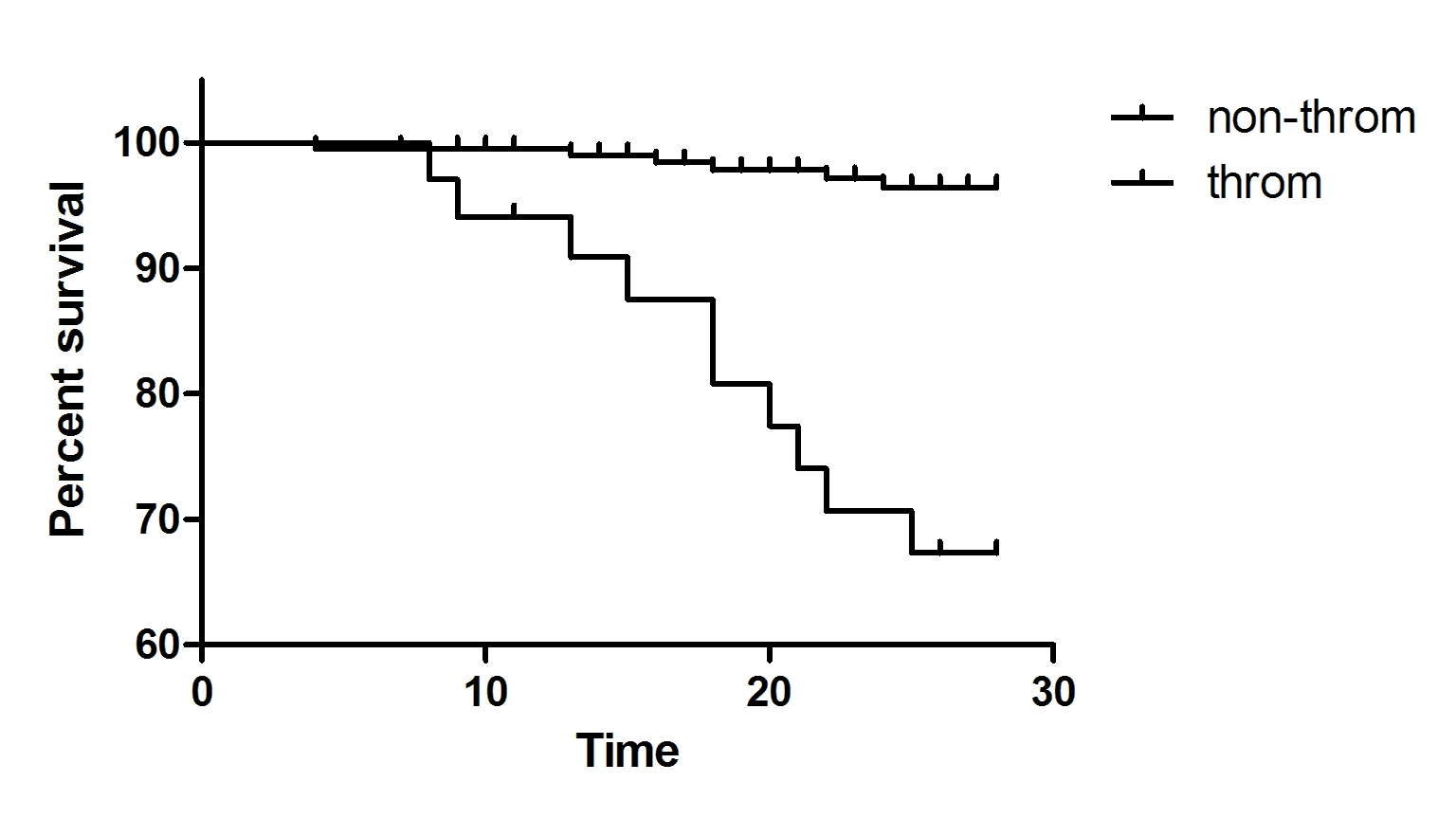

Supplement: S2 Fig — A significant difference in mortality was observed between hospital-acquired thrombocytopenia patients and non- hospital-acquired thrombocytopenia patients. (TIF) [file pone.0147482.s002.tif]
